# Lung Involvement in Inflammatory Bowel Diseases: Shared Pathways and Unwanted Connections

**DOI:** 10.3390/jcm12196419

**Published:** 2023-10-09

**Authors:** Carolina Aliai Micol Cavalli, Roberto Gabbiadini, Arianna Dal Buono, Alessandro Quadarella, Alessandro De Marco, Alessandro Repici, Cristina Bezzio, Edoardo Simonetta, Stefano Aliberti, Alessandro Armuzzi

**Affiliations:** 1 IBD Center, IRCCS Humanitas Research Hospital, Via Manzoni 56, Rozzano, 20089 Milan, Italy; carolina.cavalli@humanitas.it (C.A.M.C.); roberto.gabbiadini@humanitas.it (R.G.); arianna.dalbuono@humanitas.it (A.D.B.); alessandro.quadarella@humanitas.it (A.Q.); alessandro.demarco@humanitas.it (A.D.M.); cristina.bezzio@hunimed.eu (C.B.); 2 Department of Biomedical Sciences, Humanitas University, Via Rita Levi Montalcini 4, Pieve Emanuele, 20072 Milan, Italy; alessandro.repici@hunimed.eu (A.R.); stefano.aliberti@hunimed.eu (S.A.); 3 Division of Gastroenterology and Digestive Endoscopy, Department of Gastroenterology, IRCCS Humanitas Research Hospital, Via Manzoni 56, Rozzano, 20089 Milan, Italy; 4 Respiratory Unit, IRCCS Humanitas Research Hospital, Via Manzoni 56, Rozzano, 20089 Milan, Italy; edoardo.simonetta@humanitas.it

**Keywords:** pneumonia, airway inflammation, lung involvement, lung cancer, respiratory tract infections, drug-induced lung injury, inflammatory bowel disease, treatment

## Abstract

Inflammatory bowel diseases (IBDs) are chronic, relapsing inflammatory disorders of the gastrointestinal tract, frequently associated with extraintestinal manifestations (EIMs) that can severely affect IBD patients’ quality of life, sometimes even becoming life-threatening. Respiratory diseases have always been considered a rare and subsequently neglected extraintestinal manifestations of IBD. However, increasing evidence has demonstrated that respiratory involvement is frequent in IBD patients, even in the absence of respiratory symptoms. Airway inflammation is the most common milieu of IBD-related involvement, with bronchiectasis being the most common manifestation. Furthermore, significant differences in prevalence and types of involvement are present between Crohn’s disease and ulcerative colitis. The same embryological origin of respiratory and gastrointestinal tissue, in addition to exposure to common antigens and cytokine networks, may all play a potential role in the respiratory involvement. Furthermore, other causes such as drug-related toxicity and infections must always be considered. This article aims at reviewing the current evidence on the association between IBD and respiratory diseases. The purpose is to raise awareness of respiratory manifestation among IBD specialists and emphasize the need for identifying respiratory diseases in early stages to promptly treat these conditions, avoid worsening morbidity, and prevent lung damage.

## 1. Introduction

Inflammatory bowel diseases (IBDs), including ulcerative colitis (UC) and Crohn’s disease (CD), are chronic inflammatory disorders of the gastrointestinal tract characterized by a natural history of relapsing–remitting flares of the disease [1,2,3]. The etiology of IBD is still not clear, but complex interactions between various risks and triggering factors have been recognized, including host genetics, immunodysregulation, gut microbiota alterations and environmental factors, which lead to an abnormal and chronic intestinal inflammation [3,4].

In addition to gastrointestinal inflammation, IBD can also be associated with different extraintestinal manifestations (EIMs) that can occur in up to 50% of patients [5,6]. EIMs represent a variety of manifestations that involve many organs outside the gastrointestinal tract. They frequently affect musculoskeletal system, skin, hepatobiliary tract, and eyes, but can also, although less frequently, involve other organs, resulting in a reduced quality of life and increased morbidity or even mortality (i.e., in case of primary sclerosing cholangitis or venous thromboembolic events) [5,6].

Respiratory diseases have always been considered a rare EIM of IBD [7,8]. However, recent studies demonstrated that respiratory diseases, since asymptomatic, are frequently underdiagnosed in IBD patients [9]. Indeed, pulmonary function tests, as well as radiological and histological pulmonary reports, can be abnormal in IBD patients, also in the absence of respiratory symptoms [5,6]. Furthermore, the respiratory involvement in IBD patients can be very heterogeneous and affect both airways, parenchyma, or interstitium [5]. The pathogenesis of respiratory involvement in IBD patients remains partially understood. The common embryological origin of the respiratory and gastrointestinal systems and their shared components of the mucosal immune system may explain the pathogenesis [10,11].

Additionally, IBD patients are at increased risk of respiratory tract infectious complications, both for the vulnerability caused by the inflammatory disease itself and for the use of immunomodulators [5,6].

The aim of this narrative review is to highlight the current evidence on the respiratory manifestations in IBD patients and to discuss the shared pathogenesis, as well as the therapeutical implications with a focus on early recognition as the main strategy to avoid complications.

## 2. Pathogenesis

**Figure 1:**
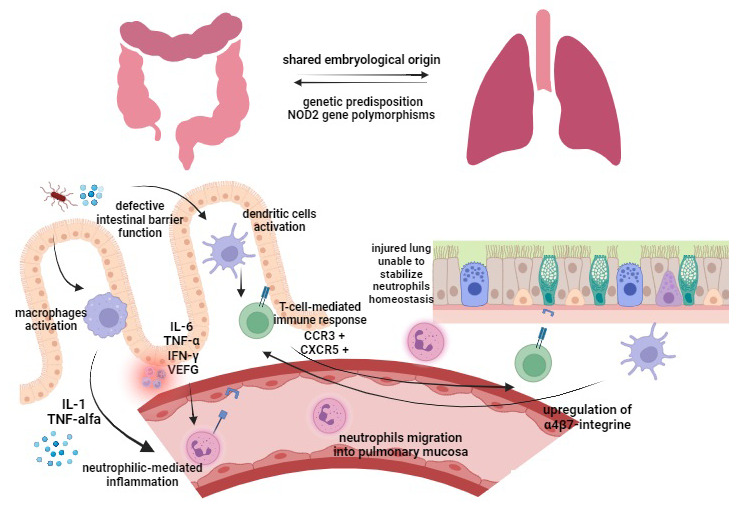
Pathogenesis of respiratory involvement in IBD patients. Gut epithelium (left) and lung epithelium (right). IL, interleukin; IFN-γ, interferon gamma; TNF-α, tumor necrosis factor; VEGF, vascular endothelial growth factor; CCR3, C-C chemokine receptor 3; CXCR5, C-X-C chemokine receptor 5.

Respiratory involvement in IBD patients can be either due to a primary extraintestinal manifestation specific to IBD (through the so-called “lung–gut axis”) or as a drug-induced adverse effect. The pathogenesis of pulmonary manifestations in IBD has not yet been explained, but some considerations have been proposed (Figure 1).

Respiratory and gastrointestinal tracts share the same embryological origin by primitive foregut, and both are characterized by epithelia with goblet cells, submucosal glands, and lymphoid tissue, which play an important role in host mucosal defense [10]. In light of these shared anatomical features, the respiratory tract may be affected by same epithelial and mucosal immune defects associated with IBD. Gastrointestinal and respiratory alterations may be due to epithelial exposure to common antigens by inhalation/ingestion (i.e., smoke, stress, infections, drugs, diet), causing sensitization and subsequent inflammation [5,10].

A defective intestinal barrier function due to chronic inflammation has been demonstrated to facilitate antigens to translocate through the intestinal epithelium [11,12,13]. Antigens passing through leaky intestinal epithelium activate both dendritic cells [14] and macrophages. Activated macrophages induce—via IL-1 and TNF-alpha—the expression of neutrophil adhesion molecules and consequently a neutrophilic-mediated inflammation [15]. Increased expression of IL-6, TNF-α, interferon-γ and vascular endothelial growth factor (VEGF) caused by bowel inflammation [16,17,18] leads to extravasation of neutrophils and increased vascular permeability in lung tissue [19,20]. Particularly, neutrophil migration into inflamed tissue takes place through processes of margination and diapedesis, which have been shown to be increased in pulmonary vasculature during systemic inflammation [21]. On the other hand, the injured lung loses its function in stabilizing neutrophil homeostasis, being unable to stop excessive neutrophil migration into the pulmonary mucosa [22]. Furthermore, the translocation of antigens through the damaged intestinal epithelium activates dendritic cells, subsequently inducing a T-cell-mediated immunoresponse [14]. Memory T-cells, which were first exposed to their specific antigen in the inflamed intestinal mucosa, have been demonstrated to bring a high number of CCR3 (C-C chemokine receptor 3) and CXCR5 (C-X-C chemokine receptor 5) [23,24]. As a result, they can translocate to the bronchus-associated lymphatic tissue (BALT), where pulmonary T-cells normally express more of these chemokine receptors. Furthermore, lung dendritic cells have been shown to be able to upregulate the expression of α4β7-integrine, leading to a T-lymphocyte migration to the gut, proving a cross link between the intestine and the lung [25].

Another molecular alteration that may play a role in both IBD and pulmonary manifestations is the dysregulation of protease activity [10,11]. During intestinal inflammation, the expression of matrix metalloproteinase (MMP) is increased, leading to a neutrophil-mediated intestinal collagen proteolysis and rise in neutrophilic inflammation [26]. Increased levels of epithelial and leukocyte MMP have already been associated with the pathogenesis of some inflammatory diseases of the lung, such as chronic obstructive pulmonary disease, and might be one cause of pulmonary manifestations of IBD [11].

Moreover, IBD and some respiratory tract diseases share variants of genes predisposing to both pathologies [27]. Particularly, NOD2 gene polymorphisms have been associated with development of both Crohn’s disease [28] and chronic obstructive pulmonary disease (COPD) [29], thus favoring the hypothesis of a common genetic susceptibility. The NOD2 receptor belongs to a family of pathogen recognition receptors (PRR), recognizing muramyl dipeptide (MDP) as part of the bacterial cell wall [30,31]. The MDP–NOD2-mediated pathway leads to an increased expression of α-defensins. Subsequently, the mutation of NOD2 can result in a diminished mucosal barrier function [32] not only in the gut but also on lung surface [11]. Moreover, an association with both asthma and Crohn’s disease was found for gene loci DENND1B, SMAD3 and SLC22A4/5 (5q31/IBD5), while the ORMDL3 gene variants present in Crohn’s disease and ulcerative colitis were also associated with childhood-onset asthma [27,33].

On the other hand, the pathogenesis of drug-induced pulmonary disease depends on the type of drug, and it can be either idiosyncratic or dose-dependent. A drug-induced bronchopulmonary toxicity should always be excluded in IBD patients receiving therapies.

Finally, opportunistic respiratory infections should be investigated in IBD patients presenting symptoms and with a history of taking corticosteroids, immunomodulators, biological therapy or small molecules.

## 3. Respiratory Tract Involvement Specifically Related to IBD

Usually, respiratory tract involvement presents from months to years after the first diagnosis of IBD [5]. Still, in up to 10% of IBD patients, respiratory involvement may be underdiagnosed because it can precede the presentation of IBD [34,35,36]. Unlike other EIMs, respiratory involvement in IBD seems not to be connected with intestinal disease activity, as it has been observed a worsening of pulmonary symptoms even after colectomy, in patients with UC [34]. Finally, every component of the respiratory system might be affected (e.g., airways, parenchyma, interstitium, vessels), leading to a polymorphic variety of pulmonary manifestations in IBD patients. In addition, subclinical pulmonary dysfunction has been increasingly described.

### 3.1. IBD-Associated Airway Diseases

Airway inflammation is the most common respiratory involvement in IBD patients (40–63% of the total of clinically significant respiratory complaints [34] from the glottis to alveolar ducts [5]), with clinical manifestations that depend on the site involved.

Cases of upper-airway disease (UAD) (including the pharynx and larynx) and proximal airway tree involvement (including the trachea and mainstream bronchi) have been described in both UC and CD patients [37]. Symptoms are cough, phlegm, hoarseness, shortness of breath, stridor, and respiratory distress. Chest examination may reveal diffuse wheezing and reduced breath sounds on auscultation. Chest X-ray is scarcely informative, mostly showing subtle findings such as tracheal narrowing, while high-resolution computed tomography (HRCT) is able to better define thickening of the tracheobronchial wall, bronchial diameter and additional findings (e.g., sputum plugs, tree-in-bud pattern) [38]. At bronchoscopy, mucosal airway inflammation shows similar lesions to those described in the digestive tract [37]. Particularly, edema and mucosal airway ulceration, deformities, whitish lesions, exuberant pseudotumoral lesions, and narrowing of the lumen resemble the mucosal lesions observed in the gut. Nodular thickening in tracheobronchitis in patients with Crohn’s disease may be the manifestation of noncaseating and epithelioid granulomas [37,38]. Histologically, biopsies reveal mucosal ulceration, infiltration by inflammatory cells and micro-abscesses, plasma-cell submucosal infiltrates, squamous metaplasia, granulation tissue; noncaseating granulomas have been reported in patients with Crohn’s disease. In the subsequent remission phase, fibrous changes appear [37]. The treatment of choice is administration of steroids (inhaled or systemic), with intravenous therapy required in life-threatening critical manifestations, such as subglottic stenosis. The risk of an untreated airway inflammation is the subsequent irreversible destruction of the airways, resulting in subglottic/tracheal stenosis. In tracheal stenosis refractory to steroid treatment, a more invasive approach may be necessary. In these cases, interventional rigid bronchoscopy (bronchoscopic dilatation, stent placement, laser beam) should be considered [39]. Surgery is indicated when the inflammation has turned into fibrosis [40].

**Figure 2:**
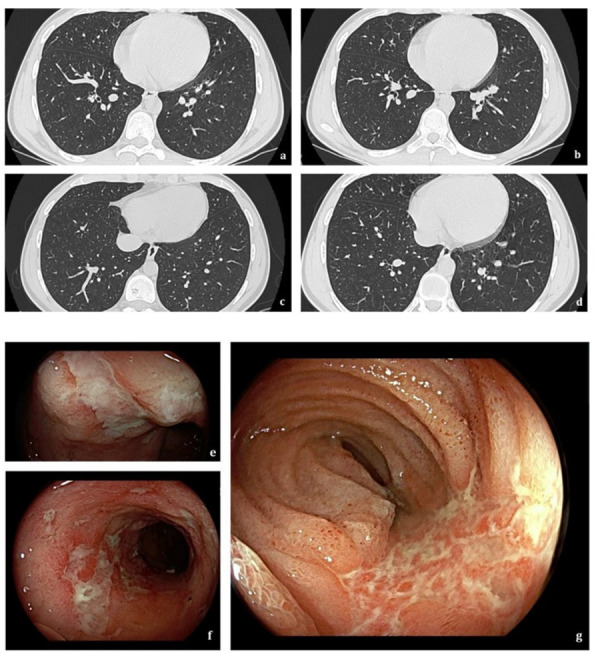
A 23-year-old man with Crohn’s disease. Axial HRCT scans (**a**–**d**) show scattered bilateral cylindrical bronchiectasis. Findings on ileocolonoscopy (**e**–**g**) include multiple ulcerations > 1 cm involving terminal ileum mucosa.

The lower airways are the most common anatomic site involved in IBD, accounting for 50% of all respiratory tract manifestations [37], with bronchiectasis being the most frequently reported, followed by chronic bronchitis and mucus impaction [5]. Lower-airway involvement is more common in nonsmoking females with UC [37,38], and it can precede IBD manifestations in younger patients (10–5%) [37]. Bronchiectasis is the most reported IBD-related respiratory tract disorder (Figure 2) [5].

Bronchiectasis is a chronic respiratory disease characterized by an enlargement of bronchi on HRCT and daily symptoms such as cough, dyspnea and sputum production [41,42]. Bronchiectasis pathophysiology is sustained by a vicious vortex composed of inflammation leading to anatomical remodeling of the airways, impaired host defenses and recurrent infections [43]. Bronchiectasis is nowadays recognized as an inflammatory disease, mainly neutrophilic but with up to 30% of patients showing a T2-high endotype [44,45,46,47,48].

Bronchiectasis appears to present more commonly in UC patients than CD, with the bowel disease usually stable or in remission. In 50% of IBD–bronchiectasis cases, a curative surgery (colectomy) preceded by weeks to months the manifestation and diagnosis of bronchiectasis [34,37,49]. This onset timing can be caused by a shift of inflammatory cytokines and mediators from the resected inflamed bowel to the lung, due to the common embryological origin of the two. Furthermore, it has been documented that IBD remission after surgery leads to a withdrawal of immunomodulatory drugs or reduction in steroids, which allows the uncovering of pulmonary disease. Antinuclear antibodies have been isolated in some of these cases, suggesting autoimmunity as a concurrent causative mechanism [37,49]. Last but not least, bronchiectasis as IBD-EIM shows more severe behavior and progression compared to other forms not associated with IBD [49].

Airway inflammation may also lead to obstructive lung diseases such as asthma, chronic obstructive pulmonary disease (COPD) or bronchiolitis.

Asthma is a chronic inflammatory lung disease characterized by expiratory airflow limitation and symptoms such as dyspnea, cough and wheeze that vary over time. It is the main pulmonary comorbidity in both UC and CD [9]. It is still not clear if the co-occurrence of asthma and IBD derives from partially shared pathogenesis (immunomediated pathways, genetic and environmental factors) or one disease results in a predisposition to the other [50]. In particular, the hygiene hypothesis has been proposed for both asthma and IBD: a lack of exposure to microorganisms during childhood can predispose to abnormal immunoreactions and subsequently chronic immunomediated diseases later in life. Furthermore, an increased risk of both asthma and IBD has been associated with exposure to antibiotics early in life, while a decreased risk has been associated with breastfeeding [50,51,52,53]. These associations suggest that a lack of exposure to enteric pathogens early in life may lead to an increased risk of developing immunomediated diseases, including both asthma and IBD [50,54,55]. IBD and asthma also share susceptibility genes, including gene loci DENND1B, SMAD3, SLC22A4/5 (5q31/IBD5) and ORMDL3 gene variants [27,33]. The coexistence of IBD and asthma increases mortality, so appropriate bronchodilator treatment and a pulmonary evaluation are mandatory [50,56].

COPD is a chronic and progressive inflammatory lung disease characterized by an irreversible expiratory flow limitation often related to cigarette smoke. Patients suffer from progressive dyspnea, cough and recurrent pulmonary exacerbations. IBDs are more frequent among COPD patients, probably due to smoking-related systemic inflammation. The coexistence of both pathologies leads to an increased mortality for all causes and specific CD-related causes [57].

Bronchiolitis is equally associated both with CD and UC. It can occur earlier in the disease course, even before the presentation of bowel disease, differentiating from airway disorders previously described. Chronic persistent bronchiolitis can progress and lead to diffuse airway narrowing and subsequent bronchiolitis obliterans syndrome (BOS). Radiological patterns include chest X-ray with normal or increased lung volumes (air trapping and/or reticulonodular or ground-glass opacities) and HRCT showing bronchiolar wall thickening, mucoid impaction, centrolobular ground-glass nodules, “tree in a bud” aspect, and mosaic due to air trapping [38]. Treatment consists of inhaled/systemic steroids and/or bronchodilators, and immunomodulators, such as TNF-alpha. Antibiotics such as macrolides, in particular azithromycin, have shown benefit in diffuse panbronchiolitis and BOS [37].

### 3.2. IBD-Associated Interstitial Lung Diseases

Lung parenchymal involvement in IBD must always raise suspicion of infections or adverse drug reaction, since specific IBD-related interstitial lung diseases are rare [34,35]. Differential diagnosis relies on clinical symptoms, pulmonary function tests, HRCT, bronchoalveolar lavage, and in some cases can require lung biopsy.

Interstitial lung involvement is more frequent in UC than CD and has a female predominance [38]. It is caused by the infiltration of the alveolar air spaces or thickening of pulmonary interstitial structures. In most cases, the development of pulmonary disease correlates with intestinal disease activity and/or other EIMs [5,58].

Several types of interstitial pneumonia have been described in IBD patients, including organizing pneumonia (OP), nonspecific interstitial pneumonia (NSIP), granulomatous interstitial lung diseases, and eosinophilic interstitial pneumonia [5].

The most common pattern of IBD-related lung parenchymal involvement is OP [5], which may also develop secondary to infection, drug toxicity, or other inflammatory disorders such as rheumatoid arthritis, lupus and Wegener granulomatosis. Histologically, an infiltration of intraluminal plugs of connective tissue in the bronchioles is observed, extending into adjacent alveolar ducts and alveoli [5]. Chest X-ray frequently displays focal/diffuse peripheral opacities and air bronchograms. HRCT is the gold standard for diagnosis, showing patchy, asymmetric foci of consolidation in a peripheral or peribronchovascular distribution, large irregular nodules, or ill-defined centrilobular nodules. Ground-glass opacities and crazy paving may also be observed. A “reverse-halo sign” (or “atoll sign”) is suggestive, although it is seen only in 20% of patients with OP [38].

NSIP is a rare manifestation in IBD. HRCT demonstrates ground-glass opacity with tiny reticulation and a subpleural and basal distribution with a sparing of the immediate subpleural lung [38].

Granulomatous interstitial lung diseases have been observed in CD patients, sharing similar characteristics to parenchymal sarcoidosis [58].

Pulmonary necrobiotic or granulomatous nodules can be difficult to differentiate from malignancies and infections. On chest radiography and HRCT, they appear as round well-defined nodules, sometimes cavitated. Usually, they respond to steroid therapy, but not to antibiotics [35,38].

Eosinophilic interstitial pneumonia has been reported in IBD patients not taking sulfasalazine or mesalamine, although most cases may be drug-induced [5,58]. HRCT patterns may show similar characteristics to OP, but with an upper-lobe predominance instead of lung bases [38].

Further rarer types of interstitial lung involvement have been also described in IBD patients, including pulmonary fibrosis with usual interstitial pneumonia (UIP) pattern, lymphocytic interstitial pneumonia (LIP), desquamative interstitial pneumonia (DIP), and hypersensitivity interstitial pneumonia (HP) [5].

### 3.3. IBD-Associated Pulmonary Embolism

Acute pulmonary embolism remains the most serious pulmonary vascular manifestation in IBD patients, since the risk of venous thromboembolism among those is at two- to threefold higher than in the general population [5,6].

Thromboembolic risk is associated with IBD pathology itself (disease activity and extent, presence of colonic disease, characteristics of fistulizing or stenosing disease) and specific related conditions, such as recent abdominal surgery or hospitalization, immobility, pregnancy, frequent central venous catheterization, and use of some drugs (such as corticosteroids or tofacitinib) [59,60,61,62]. Particularly, patients taking only corticosteroids as IBD treatment have a hypercoagulability state that leads to an increased risk of venous thromboembolism, which is five times higher than those who were treated with only biologics [63]. Conversely, anti-TNFα therapy is associated with a decreased risk of VTE by decreasing coagulation biomarkers and activates fibrinolysis [5,59,64,65].

### 3.4. IBD-Associated Vasculitis

A rare association between vasculitis and IBD has been described, predominantly from case reports and small case series. The diagnosis of IBD usually precedes vasculitis and there is a female predominance [66]. Few cases of granulomatosis with polyangiitis (GPA) associated with IBD have been described, presenting with features of systemic illnesses, nodular lung lesions, and antineutrophil cytoplasmic antibodies positivity. Lesions resolved promptly after treatment with corticosteroids. Rare cases of eosinophilic granulomatosis with polyangiitis (EGPA), microscopic polyangiitis, and other pulmonary vasculitis non-ANCA+ have been reported by some authors [66,67]. A summary of IBD-associated vasculitis and other patterns of bronchopulmonary involvement specifically related to IBD can be found in Table 1.

### 3.5. Subclinical Pulmonary Dysfunction in IBD

Pulmonary function tests (PFTs) and radiological and histological pulmonary reports can be abnormal in IBD patients, even in the absence of respiratory symptoms, suggesting the potential presence of subclinical pulmonary dysfunctions [68,69,70,71].

Ellrichmann et al. [72] found a significant reduction in respiratory function in IBD patients. IBD patients with active disease had significantly reduced FEV1 (forced expiratory volume in one second) values compared with patients in remission and with healthy controls. Interestingly, the level of pulmonary obstruction correlated with clinical IBD inflammation scores (Harvey–Bradshaw Index (HBI) for CD and partial Mayo (pMayo) score for UC patients). Peripheral airway obstruction, assessed by MEF 75–25 (maximal expiratory flow at 25%, 50%, and 75%), showed impaired values in patients with active disease but not in remission. Remarkably, only 43% of patients with active IBD had respiratory symptoms, measured with a positive Medical Research Council dyspnea score (five-graded clinical symptom score).

Diffusing capacity for carbon monoxide (DLCO)-impaired values are the most common finding in the PFTs of IBD patients [73]. Reduced DLCO values have been described in IBD patients [70,74,75,76,77,78,79,80,81,82], with some studies associating lung involvement with IBD activity [70,74,75,76,78,81,82]. Interestingly, many of the patients with PFT abnormality were free of respiratory symptoms [75,78].

The study of lung volumes and in particular of RV (residual volume), TLC (total lung capacity) and RV/TLC ratio demonstrated alterations in IBD patients, even asymptomatic [75]. Specifically, RV, TLC and RV/TLC ratio have been found to be elevated in IBD patients and to be related to disease activity [75,83].

Bronchoprovocation challenge testing showed increased bronchial hyperresponsiveness to the administration of methacholine in IBD patients with no respiratory symptoms [84,85], but no association with disease activity or duration has been found [86].

On exhaled nitric oxide (NO) measurement, increased exhaled NO has been found in IBD patients [87,88,89,90], suggesting the presence of subclinical small-airway inflammation, with a positive association with bowel disease activity [87,88,89].

This subclinical pulmonary involvement also characterized radiological findings in IBD patients. HRCT in two series of IBD patients showed pathological findings in 53% and 64% of patients, respectively [75,78], showing peribronchial thickness, air trapping, fibrosis, emphysema, bronchiectasis and alveolitis. Interestingly, these findings were independent of the presence of respiratory clinical symptoms in more than 50% of the patients. The peribronchial thickness might reflect early inflammation. In adjunction, bronchial dilatation is commonly present and results from traction by fibrous tissue on the bronchial walls, subsequently leading to bronchiectasis [36].

Interestingly, treatment with anti-TNF is associated with significantly improved obstruction (*p* = 0.003 for FEV1% in comparison with baseline levels) in both CD and UC patients, suggesting that inflammation promoted by proinflammatory cytokines may have an important role in the pathogenesis of IBD-associated pulmonary involvement [72]. This was also supported by Aydin et al., who demonstrated an alveolar hemorrhage in animal models (rats) with acute colitis, caused by significantly elevated concentration of VEFG and TNF-alpha in pulmonary tissue in induced-colitis groups compared with control rats [91].

Regular PFT screening and assessment may represent an important adjunctive investigation in IBD patients, in order to early detect latent respiratory involvement. Conflicting data are present in existing literature regarding the use of PFTs in IBD patients, and currently, no clear evidence supports routine screening for respiratory involvement in IBD patients [6]. Certainly, PFTs may have a role in identifying patients with IBDs who require further evaluation of the respiratory system. Future prospective studies are required to clarify the role of PFTs as diagnostic investigations for detecting subclinical pulmonary involvement and determining the activation of IBDs.

### 3.6. IBD-Associated Lung Cancer

The risk of intestinal and colonic cancer in IBD has been widely investigated, while less is known about the risk of extraintestinal cancer (EIC).

Perforating CD and extensive UC have been identified as risk factors for overall cancer and for extracolonic cancers in a 6-year multicenter prospective nested case–control study by Biancone et al. [92].

Regarding lung cancer, a meta-analysis by Lo et al. in 2021 showed an increased risk of lung cancer in CD patients, while in UC patients, no significant risk of was found [93]. Notably, two recent population-based studies found an increased risk of lung cancer in CD patients. In 2013, a Danish population-based IBD cohort study by Jess et al. found CD patients, but not UC, to have an increased risk of lung cancer, particularly associated with female gender and smoking [94]. In 2014, a Danish population-based cohort study with 30-year follow-up by Kappelmann et al. found an increased risk of extraintestinal malignancies in CD patients, with a strong association with smoking-related cancers, including lung, larynx, oral cavity, pharynx, bladder, kidney, and ureter (standardized incidence ratios 1.5, 95% CI 1.3–1.8) [95]. No significant risk of lung cancers in UC patients has been demonstrated in any study. Some studies found that UC patients had a significantly lower risk of lung cancer [96,97], possibly reflecting differences in smoking habits between CD and UC patients.

It is always important to carefully evaluate lung cancer risk in IBD patients, considering both risk factors related to the disease (CD phenotype, severity of the disease, immunomodulator use) and those common to the general population (smoking and second-hand smoking, exposure to radon, asbestos, and cancer-causing agents such as chromium, cadmium, arsenic, radioactivity, and coal products) [98].

### 3.7. Respiratory Infections in IBD Patients

IBD patients, particularly those treated with immunosuppression, are at increased risk of respiratory infections [99].

Risk factors for respiratory infections in this population are the use of immunosuppressive agents (particularly in combination), intestinal disease activity, malnutrition, older age, congenital and acquired immunodeficiency, chronic diseases, diabetes mellitus, total parenteral nutrition, and bowel surgery [99].

Among different microorganisms causing respiratory infections, IBD patients are at an increased risk of pneumococcal infection, with a two- to threefold higher risk of invasive pneumococcal disease (meningitis and bacteremia), even in the 5 years preceding IBD diagnosis [100]. Bacterial pneumonia is one of the most common infections in immunosuppressed patients with IBD [99,101]. ECCO guidelines recommend pneumococcal vaccination (single dose of PCV13 followed by PPSV23 after 8 weeks, and a PPSV23 booster after 5 years) for all IBD patients, since the 1-year mortality is lower in patients with IBD vaccinated against pneumococcus (2.1%) compared with those not vaccinated (4.5%) [99,101].

Bacterial pneumonia in IBD patients on immunosuppressive therapy should always be tested and excluded for presence of Legionella pneumophila. In fact, some fatal invasive L. *pneumophila* infections have been reported in patients on immunomodulators for IBD [102]. Among immunomodulators, anti-TNF agents present a major risk of L. *pneumophila* infection [102].

Other relevant respiratory infections in IBD patients are both active pulmonary tuberculosis (TB) and latent TB infection (LTBI). Screening for LTBI before starting any treatment is recommended [99], since exposure to biologic agents is associated with an increased overall risk of active tuberculosis [103]. ECCO guidelines also suggest considering annual rescreening in high-risk patients (living or traveling in TB-endemic areas), since there have been TB cases in patients exposed to anti-TNF agents, despite a negative TB screening preceding anti-TNF therapy [104,105]. In addition to anti-TNF agents, Janus kinase (JAK) inhibitors are associated with increased risk of reactivation of LTBI [99,106]. A study by Winthrop et al. evaluated tofacitinib-exposed patients and found TB to be the most common opportunistic infection, presenting with more severe and extrapulmonary disease forms than in the general population [106]. It occurred rarely in patients treated with 5 mg twice daily and in regions of low TB prevalence [106]. ECCO guidelines recommend evaluating rescreening patients previously exposed to biologic agents and JAK inhibitors before switch or swap [99]. Diagnosis of LTBI requires a complete therapeutic regimen before starting biologic agents, small molecules, or high-dose systemic steroids: IBD treatment should start at least 4 weeks after chemotherapy.

Among viruses, an increased influenza virus risk has been documented in IBD patients, who may also have more complications, firstly pneumonia, thus requiring more hospitalization [107]. ECCO guidelines recommend annual influenza vaccination of patients on immunosuppressive therapy [99]. Conversely, SARS-CoV-2 does not seem to cause more severe disease in IBD patients. Anti-TNF monotherapy, vedolizumab, and ustekinumab were not associated with severe COVID-19 [108]. In immunosuppressed IBD patients, herpes simplex virus reactivation may cause severe localized systemic infections including pneumonia, as well as measles outbreaks, which can present without rash or fever, but with life-threatening giant-cell pneumonitis.

Parasitic or fungal infections are rare in IBD patients. They should be investigated in high-risk patients (residents or traveling in endemic areas). ECCO recommend prophylaxis concerning *Pneumocystis jiirovecii* infections in IBD patients on triple or double immunosuppressive therapy (steroids, methotrexate, thiopurines, biologics), for whom standard prophylaxis with trimethoprim–sulfamethoxazole (TMP-SMX) should be strongly considered. TMP-SMX should also be considered for any combination of high-dose corticosteroids, low lymphocyte count, or JAK inhibitors [99].

Depending on the severity of the infection, temporary lengthening of the biologic interval or withdrawal of the drug until symptom resolution should always be considered.

## 4. Drug-Induced Pulmonary Manifestations

### 4.1. Salicylates (Sulfasalazine, Mesalazine)

Mesalazine (5-aminosalicylic acid, 5-ASA) represents the first-line treatment for mild-to-moderate ulcerative colitis, and it has been largely associated with drug-induced lung injury. Mesalazine-induced pulmonary reactions in IBD patients were first described in 1991 [109].

The pathogenesis of mesalazine-induced pulmonary adverse drug reactions is still not clear. It is hypothesized that mesalazine can cause both a direct, dose-dependent insult to pulmonary epithelium and an immunomediated alveolitis [110,111].

Mesalazine can induce different types of interstitial lung disease in the form of eosinophilic pneumonia, organizing pneumonia, and nonspecific interstitial pneumonia [5,7,112]. Rare cases of hypersensitivity pneumonitis have been described. Almost all cases presented with mild or no respiratory failure and were successfully treated only by discontinuation of the drug or administration of low-dose corticosteroids. Only two cases of severe respiratory failure due to mesalazine use have been described in the literature [113,114]. The time from drug exposure to lung injury is unclear from the literature (from days to months), so further studies are needed to better understand the relationship between time from drug exposure and lung injury.

Sulfasalazine is a combination of 5-aminosalicylic acid and sulfapyridine, joined by an azo bond. The sulfapyridine component acts as a carrier of the active component 5-ASA to the colon, where the azo bond is broken by gut organisms. The sulfapyridine is absorbed and subsequently excreted in the urine [115]. The exact pathogenesis causing lung toxicity is not still well known, but the sulfapyridine component is believed to be responsible for most hypersensitivity reactions that can occur [115]. The types of interstitial lung disease described are eosinophilic pneumonia (the most common), fibrosing alveolitis, and less commonly bronchiolitis obliterans and organizing pneumonia [116]. Clinical manifestations include breathlessness, fever, cough, weight loss and chest pain, and 50% of patients had a peripheral eosinophilia. Management included withdrawal of the drug and a possible addition of steroid treatment based on the severity of the adverse drug reaction. The majority of patients with sulfasalazine-induced lung disease had completely resolution in a few weeks with discontinuation of the drug [116].

### 4.2. Azathioprine and 6-Mercaptopurine

Azathioprine (AZA) is used for the maintenance of remission in IBD. Relatively common side effects are both early hypersensitivity reaction (nausea, fever, hepatitis and pancreatitis) and late bone marrow depression (leukopenia and macrocytosis). Conversely, azathioprine/6-MP-related pulmonary toxicity is a rare but serious side effect [117].

Case reports of AZA-associated interstitial pneumonia and organizing pneumonia have been described [117,118]. Patients presented with dyspnea, cough, and fever within one month after initiation of azathioprine/6-MP. Discontinuation of treatment, eventually associated with corticosteroid medication, led to clinical improvement [118].

### 4.3. Methotrexate

Methotrexate (MTX) has been associated with lung injury in the form of MTX-related hypersensitivity pneumonitis or pulmonary fibrosis. The incidence of MTX-induced pneumonitis ranges from 0.3% to 11.6% [119,120]. The pathogenesis seems to be dose-independent through a hypersensitivity reaction, usually occurring early after MTX commencement [120]. Most patients with MTX pneumonitis presented subacute onset of symptoms: shortness of breath, dyspnea, cough and fever. Peripheral blood eosinophil count is elevated in about 20% of the cases [121,122]. Some case reports in the literature have suggested a link between MTX and chronic lung fibrosis [123,124,125], but it is still not clear whether the lung damage is a consequence of the underlying disease or due to methotrexate [126]. Clinicians should be cautious when starting MTX in patients with preexisting lung disease, since lung adverse reactions are rare but critical and can lead to severe outcomes.

### 4.4. Biological Therapy

#### 4.4.1. Anti-TNF Agents

Anti-tumor necrosis factor (TNF) agents (infliximab, adalimumab, and golimumab) are recommended to induce and maintain remission in patients with moderate-to-severe UC and CD [127,128]. TNF is one of the main proinflammatory cytokines and also plays a key role in response to infection.

Some anti-TNF-induced pulmonary complications have been identified: infections (TB, bacterial and fungal infections), exacerbations of underlying lung disease, interstitial lung disease (ILD), granulomatous lung disease, systemic lupus erythematosus (SLE)-like reactions and vasculitis [129].

With respect to infections, in addition to surveillance for TB prior to initiation of TNF-targeted therapy, vigilance for infectious complications should be maintained during the therapy course, since there is a known increased risk of opportunistic infections, mostly mild forms [103].

Interstitial lung disease has been reported in association with infliximab use, although infrequently. Infliximab-induced ILD may probably be due to a CD8 T-cell mediated hypersensitivity reaction [130,131]. Perez-Alvarez et al. [132] analyzed the largest sample of patients with lung injury secondary to anti-TNF therapy between January 1990 and March 2010. They found 122 cases of ILD (58 associated with etanercept, 56 associated with infliximab, 3 secondary to adalimumab). ILD appeared approximately 26 weeks after initiation of the biologic agent in the forms of usual interstitial pneumonia pattern, organizing pneumonia, diffuse alveolar damage, and even lymphocytic interstitial pneumonia. Regarding adalimumab, some case reports of adalimumab-induced ILD have been reported [129,130,132,133,134,135,136,137,138,139]. Symptoms of adalimumab-induced ILD are dry cough, dyspnea, fever, malaise, and shortness of breath. In about 65% of cases, the withdrawal of the drug led to complete resolution.

Some increasing reports in the literature described TNF-targeted therapies causing autoimmune disease [140,141,142]. The pathogenesis leading to formation of new autoantibodies during anti-TNF therapy is not completely understood. Theoretical considerations include alteration of apoptosis with increased exposure of antigens to the immune system and B-cell activation. Anti-nuclear antibody formation has been described in 34–95% of rheumatoid arthritis patients treated with infliximab and 11–26% with adalimumab [140]. Ramos-Casals et al. described 226 patients exposed to anti-TNF-alpha who developed autoimmune disease that included vasculitis (*n* = 113), lupus (*n* = 92), and interstitial lung disease (*n* = 24) [141]. Diri et al. recently described three patients exposed to infliximab who developed lupus-like syndrome involving the lung and pleura [142].

#### 4.4.2. Ustekinumab

Ustekinumab is a fully human monoclonal antibody IL-12 and IL-23 antagonist, recommended to induce and maintain remission in patients with moderate-to-severe UC and CD [127,128].

Nasopharyngitis and upper respiratory tract infection were part of the most frequently reported adverse effects in the UNIFI long-term (156 weeks) extension [143].

Cases of pulmonary toxicity related to ustekinumab are limited. A case series by Brinker et al. [144] in 2019 identified 12 patients taking ustekinumab for psoriasis that developed respiratory symptoms within 2 years of drug initiation. The pulmonary adverse events described included interstitial pneumonia (seven patients), organizing pneumonia (one patient), eosinophilic pneumonia (three patients), and hypersensitivity pneumonitis (one patient), based on results of imaging, BAL findings, and/or lung biopsy. All cases needed medical therapies, with some of them even requiring hospitalization. Kalra et al. [145] in 2020 described a patient with Crohn’s disease presenting dry cough and dyspnea after the first dose of ustekinumab, who was subsequently diagnosed with chronic eosinophilic pneumonia based on imaging findings, negative autoimmune serology, and BAL with 67% eosinophils. Ustekinumab was withdrew and high-dose systemic steroid therapy was started, with resolution of the lung involvement. Despotes et al. [146] in 2022 described a case of acute hypoxic respiratory failure due to ustekinumab-induced lung disease in a Crohn’s patient. The patient was treated with ustekinumab 2 years prior to this event, with good response, but stopped the drug after 5 months due to concerns about potential infections. Later, ustekinumab was restarted due to a flare of active Crohn’s disease, and 2 weeks after restarting ustekinumab, he presented fever and subsequent hypoxemic respiratory failure. Infections and autoimmunity were excluded, so ustekinumab was stopped, with subsequent dramatic improvement. A drug-induced interstitial lung disease (DILD) secondary to ustekinumab was diagnosed. In 2015, the case of a 71-year-old patient with psoriasis treated with ustekinumab who developed eosinophilic pneumonia was reported [147]. In 2017, Ali et al. described the case of a 61-year-old patient treated with ustekinumab for worsening psoriasis who developed ustekinumab-induced hypersensitivity pneumonitis 5 weeks after starting therapy [148].

Generally, treatment consists of discontinuation of ustekinumab, with or without adjunction of steroid therapy.

The mechanism of ustekinumab-DILD is not fully understood. A hypothesis is that it represents a manifestation of hypersensitivity reaction. As described by Schwaiblmair et al., drugs can act as potential antigens, subsequently activating an immune cascade by drug-specific antibodies or drug-specific T cells to induce lung toxic effects [149]. Yashiro et al. [150] proposed that the inhibitory effect of ustekinumab on IL-12 and IL-23 could impede T-helper cell T_H_1 and T_H_17 activity causing a T_H_2-dominant response, thus triggering the onset of eosinophilic pneumonia.

If an ustekinumab-related DILD is suspected, it is advisable to stop the drug and to consider early high-dose steroid treatment, tapered over subsequent weeks: ustekinumab has a long half-life and could remain in the system for a prolonged period, continuing to cause damage.

#### 4.4.3. Vedolizumab

Vedolizumab (VDZ) is a fully humanized monoclonal antibody α4β7 integrin receptor antagonist, recommended to induce and maintain remission in patients with moderate-to-severe UC and CD [127,128]. It is an intestinal selective biological agent that blocks the receptor’s interaction with mucosal addressin cell adhesion molecule-1, causing inhibition of migration of T lymphocytes into the intestinal parenchymal tissue [151].

Vedolizumab therapy has not been associated with an increased incidence of respiratory tract infection in data published by Feagan et al. [152]. Conversely, a meta-analysis conducted by Marafini et al. found a significantly higher number of respiratory tract infections (RTI) in the vedolizumab-treated group than in the placebo group (for upper RTI, but not lower RTI) [153]. This finding might be due to the expression of MAdCAM-1 in the oropharynx [154] such that vedolizumab could block migration of host T cells against pathogens (e.g., CD8^+^ T cells) toward the upper respiratory mucosa.

Some cases of noninfective lung injury related to vedolizumab have been reported in the literature. Pugliese et al. [155] described the largest series of noninfective vedolizumab-related pneumonitis (*n* = 10). All ten patients developed respiratory symptoms with radiologic findings of interstitial pneumonitis after a median of four vedolizumab infusions, with full recovery after its withdrawal and steroidal therapy. The most common symptoms were cough, fever, and dyspnea. Interestingly, one patient tried to restart vedolizumab after pneumonitis resolution, but the symptoms relapsed after 2 weeks. In 2017, Sudheer et al. reported the case of a 58-year-old UC patient who developed ARDS (acute respiratory distress syndrome) after receiving induction of vedolizumab. He required intubation and mechanical ventilation. By withholding vedolizumab and giving steroid therapy, the patient was successfully treated [156]. Recently, other case reports documented the possibility of vedolizumab-induced lung injury [157,158,159].

The pathogenesis of vedolizumab lung damage has not been fully explained. The bound of vedolizumab to α4β7 and its internalization may make other integrins (such as β1) more prevalent on leukocyte surfaces: proinflammatory leukocyte homing might shift toward non-intestinal sites, including lung [160].

Cases of granulomatous lung disease [160,161] and necrobiotic pulmonary nodules in patients with Crohn’s disease during treatment with vedolizumab have been described [162]. Rare cases of eosinophilic pneumonia and eosinophilic bronchial asthma in patients with UC under vedolizumab therapy have also been described [163,164].

Hypothesis for vedolizumab-associated eosinophilic pneumonia include obstruction of VDZ-associated cells in the gastrointestinal tract, allowing for immune effector cells to spread to external intestinal sites. Alternatively, VDZ-induced eosinophilic pneumonia may be a non-IgE-mediated hypersensitivity reaction [165].

### 4.5. Small Molecules

Small-molecule Janus kinase (JAK) inhibitors comprise a group of molecules (JAK1, JAK2, JAK3, TYK2) essential to the intracellular signal cascade originating from extracellular cytokine receptors to the nuclei of immune cells. Inhibition of tyrosine kinase enzymatic activity can disrupt the activity of key interleukins.

Common side effects of this class of drugs include infections, most commonly those of the respiratory tract and an increased risk of herpes zoster. In 2020, a metanalysis by K. Khoo et al. [166] described a statistically significant increase in the incidence of upper and lower respiratory tract infections using small-molecule JAK inhibitors (smTKIs), with a major risk associated with tofacitinib. However, the risk of respiratory infections was found to be broadly comparable to that of anti-TNF agents. Particularly, smTKI treatment has been associated with a statistically significant increase in the incidence of influenza and pneumonia compared with placebo. No significant increase in risks of interstitial lung disease and lung neoplasm was documented in the metanalysis.

Conversely, in 2022, Ytterberg et al. conducted the Oral Rheumatoid Arthritis Trial (ORAL) Surveillance trial [167], aiming at evaluating the safety and efficacy of tofacitinib compared with anti-TNF in patients with rheumatoid arthritis who were 50 years of age or older and had at least one additional cardiovascular risk factor. Regarding cancer risk, during a median follow-up of 4.0 years, the incidence of cancers (excluding nonmelanoma skin cancer) was higher with the combined tofacitinib doses compared to a TNF inhibitor, with lung cancer being the most common cancer described in the tofacitinib group. Furthermore, upper respiratory tract infections and bronchitis were two of the most common adverse events, with pneumonia being the most common serious adverse event. Tuberculosis was found to be more frequent with both tofacitinib doses than with an anti-TNF.

Venous thromboembolism is another of the main themes related to small molecules, due to raised concerns in post-marketing surveillance in people treated with tofacitinib. A higher frequency of pulmonary embolism (PE) in patients receiving tofacitinib 10 mg twice daily versus those receiving anti-TNF was identified in the ORAL Surveillance trial [167]. In a post hoc analysis from the tofacitinib UC clinical development program, all deep vein thrombosis (DVT)/pulmonary embolism (PE) events occurred during the OLE study, after at least 7 months of treatment, in patients receiving 10 mg bid, and all of them had at least one venous thromboembolism risk factor [168]. In the OCTAVE Open open-label, long-term extension (up to 7.0 years) all DVT and PE events were found in the tofacitinib 10 mg bid group, although the IRs for deep vein thrombosis and pulmonary embolism were comparable with those reported for patients with ulcerative colitis in general. Particularly, in the tofacitinib 10 mg bid group, one (0.1%) patient had a deep vein thrombosis and five (0.7%) patients had pulmonary embolism. The patient with deep vein thrombosis had a history of long-haul flights and management of an infected leg wound caused by a recent accident. Four of the five patients with pulmonary embolism had a history of risk factors: prior deep vein thrombosis and pulmonary embolism; phlebothrombosis and stroke; oral contraceptives; cholangiocarcinoma with metastases [169].

## 5. Conclusions

Respiratory involvement has always been considered a rare extraintestinal manifestation of IBD, probably underestimated in daily clinical practice. Indeed, few data are available about its frequency and its relationship with intestinal disease activity.

Two main pathogenetic patterns are recognized in this association: respiratory tract manifestations specifically related to IBD and drug-induced injuries. Airway inflammation is the most common milieu of IBD-related involvement, with bronchiectasis being the most common manifestation. Specific IBD-related interstitial lung disease is a rare entity, so differential diagnosis with infections and adverse drug reactions must always be suspected. Furthermore, IBD patients present a vulnerability to infections due to disease activity itself and by use of immunomodulator and immunosuppressant drugs. On the other hand, drug-related lung toxicity must be always ruled out because it is the most common pulmonary manifestation of IBD.

Therefore, it is important to emphasize the need for identifying IBD-related respiratory diseases in early stages to promptly treat these conditions, avoid worsening morbidity and prevent lung damage. For these reasons, it is important to appropriately screen patients at the diagnosis of IBD and before the start of treatment with pulmonary medical history, clinical examination, and microbiological and radiological tests. A close follow-up of IBD patients and early pneumologist consultation during treatment is essential to evaluate adverse reactions. Appropriate and early management of a drug-induced injury prevents progression to respiratory failure or other serious outcomes.

## Figures and Tables

**Table 1:** Bronchopulmonary involvement in inflammatory bowel diseases.

**Anatomical Site**	**Pathology**	**Symptoms**	**Imaging and Additional Investigations**
Airways diseases	▪ Glottic and subglottic edema/stenosis ▪ Isolated tracheal inflammation/bulging ▪ Tracheobronchitis ▪ Bronchiectasis ▪ Mucoid impaction ▪ Diffuse panbronchiolitis ▪ Granulomatous bronchiolitis ▪ Bronchiolitis obliterans syndrome ▪ Asthma ▪ COPD	Cough Phlegm Hoarseness Dyspnea Stridor Respiratory distress Sputum production Wheezing	▪ Chest X-ray ▪ HRCT ▪ Bronchoscopy ± biopsy ▪ Pulmonary function tests including plethysmography, DLCO and bronchoreversibility ▪ Sputum cultures ▪ Referral to pulmonologist
Interstitium	▪ Organizing pneumonia ▪ Non-specific interstitial pneumonia ▪ Granulomatous interstitial lung diseases ▪ Eosinophilic interstitial pneumonia ▪ Usual interstitial pneumonia ▪ Lymphocytic interstitial pneumonia ▪ Desquamative interstitial pneumonia, ▪ Hypersensitivity interstitial pneumonia ▪ Pulmonary necrobiotic or granulomatous nodules	Dyspnea Fever Acute respiratory failure Chest pain Dry cough	▪ Chest X-ray ▪ HRCT ▪ Pulmonary function tests including plethysmography, DLCO and bronchoreversibility ▪ 6 min walking test ▪ Referral to ILDs clinic ▪ Lung biopsy
Pulmonary embolism	▪ Pulmonary embolism	Cough Palpitations Chest pain Shortness of breath	▪ If acute symptoms, send to Emergency Department ▪ CTPA ▪ Echocardiography ▪ Blood exams ▪ Arterial blood gas analysis ▪ Referral to pulmonologist
Vasculitis	▪ Granulomatosis with polyangiitis (GPA) ▪ Eosinophilic GPA (EGPA)	Cough Shortness of breath Constitutional symptom (fever, weight loss, or fatigue)	▪ Chest X-ray ▪ HRCT ▪ Pulmonary function tests including plethysmography, DLCO and bronchoreversibility ▪ 6 min walking test ▪ Blood exams including autoimmunity ▪ Referral to pulmonologist and/or rheumatologist ▪ Lung biopsy

COPD: chronic obstructive pulmonary disease; HRCT: high-resolution computed tomography; DLCO: diffusing capacity of the lungs for carbon monoxide; ILD: interstitial lung disease; CTPA: computed tomographic pulmonary angiography.
